# Running away from phonological ambiguity, we stumble upon our words: Laboratory induced slips show differences between highly and lowly defensive people

**DOI:** 10.3389/fnhum.2023.1033671

**Published:** 2023-03-29

**Authors:** Lola Thieffry, Giulia Olyff, Lea Pioda, Sandrine Detandt, Ariane Bazan

**Affiliations:** ^1^Laboratoire InterPsy (UR 4432), Université de Lorraine, Nancy, France; ^2^Observatoire du Sida et des Sexualités, Université Libre de Bruxelles, Brussels, Belgium; ^3^Parhélie Asbl, Institution Psychiatrique, Brussels, Belgium; ^4^Faculté des Sciences Psychologiques et de l’Éducation, Université Libre de Bruxelles, Brussels, Belgium; ^5^Centre de Recherche en Psychologie Clinique, Psychopathologie et Psychosomatique, Université Libre de Bruxelles, Brussels, Belgium

**Keywords:** Freud, slips of the tongue, repression, defense, metapsychology, unconscious, preconscious, signifier

## Abstract

**Introduction:**

Freud proposed that slips of the tongue, including apparently simple ones, always have a sense and constitute « a half-success and a half-failure » compromise resulting from defensive mechanisms.

**Material and methods:**

A total of 55 subjects participated in a French adaptation of the *Spoonerisms of Laboratory Induced Predisposition* or SLIP-technique including 32 “neutral” and 32 taboo spoonerisms and measures of defensiveness. In accordance with a psychoanalytical and empirically supported distinction, we considered two kinds of defenses: elaborative or primary process and inhibitory or secondary process defenses, which were operationalized with the GeoCat and the Phonological-Nothing (PN) WordList, respectively. The GeoCat is a validated measure of primary process mentation and the PN WordList was shown to measure the defensive avoidance of language ambiguity.

**Results:**

Participants produced 37 slips, with no significant difference in the number of “neutral” and taboo slips. The GeoCat and the N/PN parameters explained 30% of the variance in the production of parapraxes, confirming the defensive logics of slips. When dividing the population into lowly and highly defensive participants (with the Marlowe Crowne Social Desirability scale), primary process mentation appears as a baseline default defense, but only highly defensive participants mobilize an additional inhibitory secondary process type of defense. Taking into account the *a priori* difference between taboo and “neutral” parapraxes, highly defensive participants made 2.7 times more taboo parapraxes than lowly defensive participants. However, if “neutral” parapraxes in both subgroups followed the same logic as the total group of parapraxes (significant contribution of primary process mentation in lowly defensives and of primary and secondary process mentation in highly defensives), these measures had no contribution to explain the occurrence of taboo parapraxes.

**Conclusion:**

We propose that Motley et al.’s prearticulatory editor, ensuring the censorship over taboo parapraxes, is an external instance of inhibition, proximal to uttering, equivalent to the censorship between the systems Preconscious and Conscious in Freud’s metapsychology. By contrast, the defenses measured in this research are internal, intimate control systems, probing for the censorship between the systems Unconscious and Preconscious, this is, for repression. This study contributes to support a psychodynamic explanatory model for the production of parapraxes.

## 1. Introduction

A little ad in *The Andover Townsman*, the hometown newspaper of the American city of Andover, on January 3, 1913 (p. 7) goes as follows: « *Mr. Spooner (…) was very shy and would never have had the courage to ask a woman to be his wife, but one afternoon, in a friend’s drawing room, he was requested to ask one of the ladies present to make tea. In doing so, he blundered as usual: “Will you take me?” he said, instead of “Will you make tea?.” Blushing, the lady “took him” and thus he “blundered” into a happy marriage* ». William Archibald Spooner (1844–1930) was an English clergyman, known for making spoonerisms frequently, so much that the word “spoonerism” is in fact derived from his last name. Spoonerisms (in French, *contrepèteries*) typically occur when transposing corresponding sounds or phonemes in words and are to be considered as a special case of slips of the tongue (Motley and Baars, 1976a). Slips of the tongue, or parapraxes, are an intriguing behavioral phenomenon dividing the field of psychology. In *Psychopathology of Everyday Life*, Freud (1901/1978, p. 271) assumes that daily life failures, such as forgetting names and words, verbal parapraxes, reading and writing errors, are not fortuitous but « have a hidden motivation ». A conflictual dynamic lies at the heart of these parapraxes which is, at least by some aspects, unacceptable to conscious thought and against which we defend ourselves. However, this defense only succeeds incompletely and will therefore give itself away (partially). For example, Freud (1901/1978, pp. 276–277, p. 279, Italics added) states that they bear « *to the greater or lesser degree (…) the marked character of being “repressed”* » and « can be traced back to *incompletely suppressed psychical material*, which, although pushed away by consciousness, has nevertheless not been robbed of all capacity for expressing itself ». This interpretation of verbal slips has become quite popular in the general public, so much so that it has given rise to the French expression *lapsus révélateurs* or “revelatory parapraxes.”

At the metapsychological level, slips of the tongue appear as an opportunity for the “system Unconscious (Ucs)” to transcend the two censors of the first topic, i.e., to get invested by the preconscious and thereupon to find an exceptional direct access to the “system Conscious (Cs)” and to voluntary motility, leading the subject to suddenly get a direct hear of his proper unbearable thoughts, and often creating embarrassment. However, psycholinguists both in Freud’s time (e.g., Meringer and Mayer, 1895) and nowadays (e.g., Rossi and Peter-Defare, 1998) consider parapraxes as accidental speech errors caused by linguistic and cognitive mechanisms and dismiss the idea that slips could be caused by repressed thoughts. Indeed, psycholinguistically, such errors are seen as failures of error control systems: while the selection of words progresses in spoken language production, either self-monitoring systems control systems (in Levelt’s serial model; e.g., Levelt, 1989; Levelt et al., 1999) or feedback dynamics (in Dell and Reich’s connectionist model; Dell and Reich, 1980, 1981) are supposed to correct for possible production errors. Moreover, in the connectionist model (Dell and Reich, 1980, 1981) word selection is subject to influences through associative priming by preceding words and to neighborhood activation mechanisms. In fact, this way of considering slips is not too far from Freud’s line of thought when he says: «… the positive factor favoring the slip of the tongue (the uninhibited stream of associations) and the negative factor (the relaxation of the inhibiting attention) invariably achieve their effect in combination, so that the two factors become merely different ways of regarding the same process. What happens is that, with the relaxation of the inhibiting attention–in still plainer terms, as a result of this relaxation–the uninhibited stream of associations comes into action » (Freud, 1901/1978, p. 61). The “uninhibited stream of associations” might be seen as quite equivalent to the spreading activation in a connectionist model and psychoanalytically amounts to primary process mentation (see further), while the “inhibiting attention” might be seen as equivalent to either the retroactive feedback mechanisms in the connectionist model or as the self-monitoring module in the serial language production model, and psychoanalytically as secondary process mentation (see further). Conceived as such, “the two factors” (spreading activation and inhibition) can be conceived of as the complementary activation of primary and secondary process mentation.

Despite these commonalities between the psycholinguistic and the psychodynamic model, deep divergences remain. Indeed, Freud (1916–1917/1966. p. 44) is keen to specify that parapraxes are not the result of a subject-less mechanism: « They are not chance events but serious mental acts; they have a sense ». In most cognitive models, the basic stages of language production, including the phonological preparation, are considered as automatic stages (e.g., Levelt, 1989), i.e., as subject-less (« without the necessity for active control or attention by the subject »; Schneider and Shiffrin, 1977, p. 2). However, Freud obliges us to suppose an intentional subject already at the level of the “basic” activation and inhibition-mechanisms underlying the production of language. This influence of the subjective structure–most patently, anxiety, and defensiveness–indeed explained results in subliminal linguistic priming research (Klein Villa et al., 2006; Bazan et al., 2019a): only anxious subjects showed subliminal palindrome priming and only defensive subjects showed subliminal aversion for phonological ambiguity. According to Shevrin (1992) personality factors do not influence cognitive tasks at the conscious level but could do so at an unconscious level.

This is also what results from psycholinguistic research on parapraxes. Indeed, verbal parapraxes can be induced experimentally with the *Spoonerisms of Laboratory Induced Predisposition* or “SLIP”-technique (Motley and Baars, 1976a). The SLIP-task asks participants to silently read word couples with shared phonemes, thereby sometimes reading out loud certain target-cued pairs. These target pairs, for example, “*balm peach*,” are designed to produce spoonerisms such as: *balm peach* → *palm beach* and are therefore spoonerism eliciting pairs (from here simply called “eliciting pairs”). This means that by interchanging the initial consonants, a new pair of word emerges which differs in meaning with the target pair. Typically, eliciting pairs are preceded by several phonological interference word pairs that resemble the phonology of the expected spoonerism and increase the probability of producing a slip of the tongue (Motley and Baars, 1976a). For example, *barred dorm* and *bought dog* precede the target *darn bore* that erroneously could be uttered as *barn door*. Motley (1985) claimed that these artificial slips of the tongue are similar to these occurring naturally. Motley et al. (1981a,1982) also have demonstrated that participants intercept taboo errors more often than neutral ones proposing that an « automatic » and « subconscious » (Motley et al., 1979, p. 196) prearticulatory component of speech production “censors” the overt formulation of taboo spoonerisms because of their socially inappropriate character. Interestingly, it has led these psycholinguistic authors, as well as others after them (e.g., Severens et al., 2011, 2012; Wagner-Altendorf et al., 2020), to make an intuitive distinction between “taboo” (e.g., *tool kits* → *cool tits*) and “neutral” (e.g., *darn bore* → *barn door*) parapraxes.

A taboo designates « something that is not acceptable to say, mention, or do on grounds of morality or taste » (Webster’s Dictionary). Taboo words designate more widely themes having to do with sexuality, death, racism, bodily productions, insults, etc. (Jay et al., 2008) and are distinct from words that “simply” have negative emotional valence (Jay, 1999; Hansen et al., 2019). During childhood, taboo words are recognized as such, through education and socialization, their use being repressed by care and authority figures (Jay, 2009). Independently of the SLIP-methodology, a number of psycholinguistic studies including taboo words (Hartsuiker and Kolk, 2001; Dhooge and Hartsuiker, 2011; White et al., 2017) have corroborated the existence of a “verbal self-monitor” which would both identify and correct errors during the speech production process and thwart the highly embarrassing utterance of taboo words, simultaneously slowing down their response times.

The Motley et al. (1981a, 1982) finding of fewer taboo than neutral slips, has thus been interpreted as the result of a prearticulatory editor, which censors the taboo slips before they are uttered. This suggests that neutral slips, in contrast, would be more likely the result of failures in the word production system. However, this is at odds with Freud, (1901/1978, p. 83), who is quick to underscore that « *even apparently simple slips* of the tongue could be traced to interference by a half-suppressed idea that lies outside the intended context » (Freud, 1901/1978, p. 83, Italics added) and: « In contrast to these groups of cases, in which the parapraxis itself brings its sense to light, there are others in which the parapraxis produces nothing that has any sense of its own, and which therefore sharply contradict our expectations. If someone twists a proper name about by a slip of the tongue or puts an abnormal series of sounds together, these very common events alone seem to give a negative reply to our question whether all parapraxes have some sort of sense. Closer examination of such instances, however, shows that these distortions are easily understood and that « *there is by no means so great a distinction between these more obscure cases and the earlier straightforward ones* » (Freud, 1916–1917/1966, pp. 41–42, Italics added). In other words, these “neutral” slips are not to be considered as “system glitches”; as a matter of fact, their target (the slip) would be precisely aimed at. It is clear that for Freud there are no *a priori* distinctions between taboo and so-called “neutral” parapraxes. Indeed, when we examine specific examples of speech blunders, they often include errors on words which acquire an emotional significance in the given context, but which outside this context would undoubtedly be deemed “neutral”. For example, in Motley et al. (1982), the potential spoonerism *darn bore* → *barn door* is said neutral. However, Klein Villa et al. (2006) recount the following anecdote: an audience member at a conference who intends to communicate that he would like the door closed in order to better hear the speaker, but is simultaneously distracted due to an inner state of boredom, states « close the bore » instead of « close the door ». We see here that this slip reveals the transgression of a taboo, namely, insulting someone. For all these reasons, there is no *a priori* ground to reject the idea that every parapraxis also has a defensive component. In summary, our first hypothesis is that ***(1) the production of parapraxes is always a defensive process, both for so-called neutral and taboo-parapraxes***.

A specific difficulty is the measurement of defensive processes. We propose that, in line with what Freud (1895/1966) describes in his *Project*, mental processes are defensive by essence: this is their ontological principle. The very reason why a mental system arises is to ward off accumulating stimulation, which threatens to burn (the membranes of) the organism; moreover, a mental apparatus grows in complexity in order to simultaneously directly discharge excess excitation and retain a fraction for the more elaborate execution of specific actions. The primary process entails the flight for incoming excitations by the shortest pathway possible: this neuronal dynamic organization is reflected in associative thinking, ruled by the pleasure principle and the overall outcome is a search for perceptual identity (Freud, 1900/1955). This perceptual identity involves the recognition and the identification of similar or identical elements that have only a fragment or attribute in common–that is, « superficial » (Freud, 1900/1955, p. 597) or « non-essential » (Holt, 1967, p. 334) similarities. However, when it comes to our internal needs (e.g., hunger) fleeing the stimulus is of no avail, and the « removal of the stimulus is only made possible here by an intervention which (…) calls for an alteration in the external world (supply of nourishment, proximity of the sexual object), which, as a specific action, can only be brought about in definite ways. » (Freud, 1895/1966, p. 316) in order to ward off this threatening tension. For this to happen, the organism « must put up with (maintaining) a store of Q (quantities of excitation) sufficient to meet the demand for a specific action » (Freud, 1895/1966, p. 297). This is the secondary process, which also inhibits primary process associative reactions (Freud, 1895/1966). In other words, a human mental system develops both primary process defense, the direct mirror-like discharge and secondary process defense, a more organized discharge also involving the inhibition of the primary process discharge. In this regard, primary and secondary process dynamics are simultaneously constitutive and defensive, be it with another functional principle (see also Bazan, submitted^1^).

This distinction is parallel to Erdelyi’s (2006) proposition who, in his *unified theory of repression*, also divides repression into two subclasses, either additive and elaborative or subtractive and inhibitory. « In inhibitory repression », Erdelyi (2006, p. 502) says, « the consciousness-lowering operation is readily conceived of as some type of psychological subtraction that results in lower consciousness (e.g., we subtract attentional allocation from a channel, we reduce or eliminate thinking about some material) ». He underscores that Freud’s initial conception of repression was of the inhibitory or subtractive variety. Freud, (1900/1955, p. 599, p. 601) literally says: the secondary process « succeeds in *inhibiting* this discharge (from the primary process) » and refers to « the inhibition imposed by the second system as the “secondary process” ». Saraga and Gasser (2005, p. 111) indicate that Freud underscored the importance of this inhibition as being the essence of the secondary process. Scano (2007, p. 141) says: « defense (…) functions by inhibiting the primary process and progressively establishing the secondary process ». For these reasons, the secondary process, which is an inhibitory type of defense, is of the kind which Erdelyi had in mind, this is, in essence, repression (see also further). Indeed, we have argued elsewhere that repression is a special instance of inhibition for highly invested *linguistic* stimuli (Bazan, 2012, p. 13). Articulation is also a « specific action » (see Bazan, 2007). For example, d’Epinay (2003, p. 88, our translation) says: « In the Project, Freud considers that this “innervation of speech” is originally a discharge mechanism, a safety valve ensuring a temporary and partial decrease in tension, along non-specific pathways, until the discovery of the “specific action” ». Specifically, this inhibition is possibly instantiated physiologically by the efference-copy-mediated attenuation of predictable proprioceptive return of the *articulation of the linguistically* grasp on stimuli (see also Bazan and Snodgrass, 2012): briefly, when initiating an action of will we predict the sensorimotor repercussions on the proper body of that action (the new positions of the muscles, the joints and the skin once the command will be carried out) and on the basis of this prediction, by anticipation, attenuate the sensorimotor cortices to that predicted level so that when the stimulation indeed comes, the feeling is readily neutralized. We have proposed that this mechanism is also the mechanism by which we attenuate the hearing of innuendos, ambiguities, and peculiar associations (see also Haskell, 1991, 2001),^2^ i.e., that it contributes to the mechanism of repression (Bazan, 2012). Erdelyi (2006) further underscores that the inhibitory-type of repression results in rebound phenomena, this is the return-of-the-repressed. In our linguistic model of repression (Bazan, 2012), unconscious inhibition of the specific articulatory action would induce return of the same *articulatory* fragments–mostly, however, as radically different meanings (e.g., the “rat obsession” in the Ratman, etiologically linked to *Heiraten* and *Frau Hofrat*), which is, of course, a radically efficient way of masking (Bazan, 2007, 2012), and thus of circumventing censorship. Specifically in parapraxes, the slips of the tongue appear as ideal ways for the return-of-the-repressed after an inhibitory kind of defense, i.e., after repression. For all these reasons, repression and the secondary process–involving the specific act of speaking and its inhibition–are logically equivalent, and of the inhibitory defense type.

For the elaborative kind of defenses, Erdelyi (2006) quotes rationalization, projection, reversal, displacement, and symbolization. Displacement is undoubtedly a primary mental process (Freud, 1900/1955) and so are symbolization (Rapaport, 1951, p. 694) and projection (Rapaport, 1951, p. 690). But for reversal too, this is obviously the case: « reversal, *or turning a thing into its opposite*, is one of the means of representation most favored by the dream-work (…) » (Freud, 1900/1955, p. 327, Italics added). In other words, reversal appears as a mirror-wise equation of one line of thought by another, which is a typical primary process fashion of mental processing. And this is true for rationalization, too; e.g., « Here is an example of (…) [an] attempt to derive one symptom from another by means of an intellectual rationalization: it is suggested that the patient, who, owing to a primary disposition, believes that he is being persecuted, infers from this persecution that he must be someone of quite particular importance and so develops megalomania » (Freud, 1916–1917/1966, p. 424). But rationalization as a defense mechanism is not limited to delusions: Freud understands rationalization as an operation that fulfills functions in the mental life serving the pleasure principle and independently of its degree of truth (e.g., Freud, 1914/1964, p. 52; Freud, 1933/1966, p. 542). Obviously, this puts rationalization under the banner of primary process defense mechanisms. Undoubtedly, then, the defense mechanisms, characterized by Erdelyi as the “elaborative kind,” function on the primary process mode. Indeed, in schizophrenia we observe primary process mentation as a defense in a straightforward way when, upon having to deal with an unexpected, and therefore “intrusive,” stimulation, a subject decompensates in delusional attribution of meaning. Our own patient Hervé (Bazan, 2012, p. 5) develops associative train thoughts on a primary process mode when intruded by visual stimuli he could not predict.

For all these reasons, we support Erdelyi’s dichotomous division of defense mechanisms in either elaborative or inhibitory dynamics and understand it as the division in primary and secondary process defense mechanisms. When it comes to parapraxes specifically, we are backed up by Freud, (1901/1978, p. 61), who proposes that parapraxes can be the result both of associative speech production (« the uninhibited stream of associations » or “positive” primary process defense) but also as the return of speech fragments which were previously put under inhibition, i.e., under tension–in other words as the return-of-the-repressed (« the relaxation of the inhibiting attention »). If we agree upon this perspective, we may now propose measures for primary process mentation and for secondary process inhibition, respectively.

For primary process mentation, the Geometrical Categorization Task (or GeoCat; Brakel et al., 2000) is a validated measure (for review see Bazan and Brakel, 2023). The GeoCat is a simple, non-verbal tool which maps preferential mobilization of primary versus secondary processes in the treatment of mental stimuli by asking participants to make similarity judgments between geometrical figures. The theoretical background is a cognitive theory of categorization that distinguishes between attributional (ATT) and configurational (REL for “relational”) similarity judgments (Smith and Medin, 1981; Murphy and Medin, 1985; Medin et al., 1990). Attributional similarity refers to the superficial resemblance between attributes of the stimuli, which is indeed the associative logic of the primary process. The specific type of relational similarity in the GeoCat is configurational with the same spatial arrangement of the components of both stimuli. Indeed, the secondary process, thanks to the “store of excitations,” which constitutes a third point, enables perspective-taking, giving access to spatiotemporal distinctions (Bazan, 2007; see text footnote 1) and this fits well with the identification of configurational similarity between stimuli in the REL items. Hence, primary process mentation is thought to be probed by the number of “attributional choices” (or ATT) and secondary process mentation by the number of “relational choices” (or REL).

As concerns the measure of the secondary process, as the GeoCat asks for forced choices between ATT and REL, the REL-choices are not independent secondary process measures. The GeoCat has in the past proven especially interesting to probe for primary process mentation (e.g., Bazan et al., 2013, 2019b). But primary and secondary process dynamics are not mutually exclusive, since they operate conjointly (e.g., Green, 1995) and are supposed to concur into the production of parapraxes. Therefore, an independent measure, specifically targeted upon linguistic inhibition, is needed here. In two previous instances in our research, we have productively made use of such a measure, namely the so-called “Phonological-Nothing WordList.” In the PN WordList, participants are presented with a prime word (e.g., *Nice -/*naıs/) and are asked to make a similarity forced-choice between a Phonological (P) target (namely, a phonological inverse, e.g., *Sign -/*saın/) and a Non-related (N) related target (e.g., *Belt*). Previous results have shown that choosing N in PN can also be understood as a *negative* choice for P: participants were thought to choose P (also) because they try to escape from phonological ambiguity (Bazan et al., 2019a). Thus, this negative choice for P would show a defensive move indicating an aversion for phonological ambiguity. This was confirmed in subliminal presentation by two independent measures of social desirability, the Marlowe Crowne Social Desirability scale (MCSD) and Balanced Inventory of Desirable Responding (interpreted as measures of defensiveness, see further), which predicted both this avoidance from phonological ambiguity on the WordList (*r* = −0.51; *p* = 0.004) as well as a neural evoked potential response, the PMN or Phonological Mismatch Negativity, interpreted as « perplexity in the face of phonological ambiguity » (Bazan et al., 2019a, p. 11). In recent results (Olyff and Bazan, 2023), a relative preference for N in PN predicts worse supraliminal rebus resolution scores. We interpreted the lesser rebus resolution in high N-PN subjects not merely as the result of a lesser phonological awareness or appetite (there were not more P-choices in a parallel Phonological-Semantic WordList), but as the result of a defensive avoidance of the ambiguous P-version of the target. For all these reasons, if the production of parapraxes is a defensive process, the number of ATT-choices in the GeoCat, as well as the number of N choices in the PN WordList made by the subject, should significantly predict the number of parapraxes produced by the subject.

Going back to Shevrin’s (1992) idea that personality plays a role at the level of operations which are deemed “automatic,” and therefore “mindless,” at a cognitive level, our second hypothesis is that personality, and more specifically, defensiveness will play a role in the production of parapraxes. Indeed, even if we think that in neurosis repression is the basic defense mechanism, we still think that neurotic persons can be lowly or highly defensive, with the level of defensiveness as a psychodynamic functioning mode, or in other terms, as a “character trait”–even if not necessarily a stable one (since people can change, e.g., through an analysis). Based upon clinical (Marin, 2011; Marmursztejn, 2013) and empirical data (Bazan et al., 2019a; Olyff and Bazan, 2023), we suppose that highly defensive people will mobilize stronger defenses, and that they will especially mobilize stronger defenses against ambiguous linguistic materials. For these reasons, we predict that ***(2) highly defensive people will produce more parapraxes than lowly defensive people***. As for the operationalization of this hypothesis, defensiveness is measured with the Marlowe-Crowne Social Desirability scale (MCSD). The initial intent of this scale was to measure social desirability, i.e., the need to present oneself in a socially desirable way (Crowne and Marlowe, 1960). Typical items ask participants to respond to behaviors that are « culturally sanctioned and approved but which are improbable of occurrence » (Crowne and Marlowe, 1960, p. 350) as for example « There have been occasions when I took advantage of someone ». It assesses to what magnitude subjects can accept undesirable but nevertheless universal and undeniable truths about themselves. However, the authors considered that their scale construct also measured defensiveness (Crowne and Marlowe, 1964, p. 206) as a « personality variable in its own right ». Indeed, « such favorably biased self-appraisal [has] to entail vulnerability in self-esteem and the use of repressive defenses » (Crowne and Marlowe, 1964, p. 206). The MCSD has since been widely used to assess defensiveness (see for example Weinberger et al., 1979; Weinberger, 1990; Eysenck and Van Berkum, 1992; Mann and James, 1998; Bazan et al., 2019a). Given that clinically, high defensiveness is especially identified through restrictive, inhibited behavior and the « use of repressive defenses » (Crowne and Marlowe, 1964, p. 206), and given our previous results in subliminal research showing a high correlation between subliminal N/PN and MCSD, we are furthermore inclined to predict that ***(3) highly defensive people will mobilize especially inhibitory-type defenses for the production of parapraxes.***

Finally, in line with previous research with the SLIP-task, ***we will also (4) explore if the a priori difference between “neutral” and taboo parapraxes is relevant in the present study***. First, are the empirical data confirming the validity of this *a priori* distinction? But furthermore, and independently of this confirmation, will highly defensive people produce more taboo parapraxes specifically? Will inhibitory defense parameters better predict taboo than “neutral” parapraxes?

In summary, our main interest for the present research is to show and start to unravel the defense mechanisms underlying the production of parapraxes.

## 2. Materials and methods

### 2.1. Participants

#### 2.1.1. Main study

A total of 55 psychology students from the *Université libre de Bruxelles* took part in the study and received course credit for participation. They had a mean age of 20 (range 18–26, *SD* = 1.8); 47 were women and one participant declared a non-binary gender identity. All participants were fluent French speakers and 40% of them were multilingual. None of these demographic variables significantly explained the production of slips.

#### 2.1.2. Variable evaluation studies

A total of 120, resp. 958 participants, recruited by advertisements on social media, took part in two online independent studies, on item tabooness (the degree to which the participants evaluated the word pair as “taboo”), resp. MCSD evaluation. They had a mean age of 32.55 (range 17–79, *SD* = 12.5), resp. of 27.52 (range 18–90, *SD* = 9.3). 81.1%, resp. 83.6%, of the sample were women and 1.1%, resp. 1.8%, declared a non-binary gender identity. A total of 43.9%, resp. 45.5%, of the participants were multilingual.

### 2.2. Materials

#### 2.2.1. The French SLIP-task

We implemented and presented Motley et al. (1981a,1982) SLIP-protocol using PsychoPy open-source software (Peirce et al., 2019). A total of 64 French spoonerisms were constructed: 32 taboo [e.g., *seau bain* → *beau sein* (*bat bucket* → *nice boob*)] and 32 neutral [e.g., *mauve phare → fauve mare* (*purple lighthouse → wildcat pond*)]. The “tabooness” of both word pairs implied in the spoonerism–the eliciting pair, and the spoonerism itself–were evaluated by 120 participants on a 7-point Likert scale (1 = not taboo - 7 = extremely taboo) in an independent online questionnaire study. The taboo eliciting pairs were more taboo (tabooness = 1.5 ± 0.9) than their neutral counterparts (tabooness = 1.3 ± 0.7; Student’s *t*-test; *p* < 0.001), and the actual taboo spoonerisms were also more taboo (tabooness = 3.3 ± 1.5) than the neutral spoonerisms (tabooness = 1.3 ± 0.8; Student’s *t*-test; *p* < 0.001). Moreover, the taboo spoonerisms were more taboo than their corresponding eliciting pairs (Student’s *t*-test; *p* < 0.001), but this is not the case for the neutral spoonerisms and their corresponding eliciting pairs (Student’s *t*-test; *p* = 0.285). Each target word pair was preceded by three phonological prime pairs constructed using Motley et al. algorithm (see Motley et al., 1982). These prime pairs are phonologically closely similar to the intended slips [e.g., *faune mâche* (*fauna salad*) for the spoonerism *fauve mare* (*wildcat pond*)].

**FIGURE 1:**
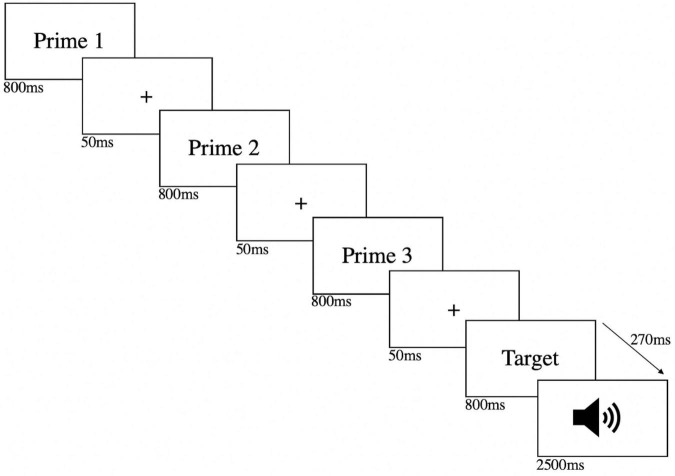
Schematic illustration of an experimental trial in the SLIP-task. Participants are shown three phonological primes before being presented with a target-cued pair (i.e., the spoonerism eliciting-pair) they have to read out loud.

An experimental trial thus consisted of three phonological primes and a target-cued or spoonerism eliciting pair (see also Figure 1). Overall, there were 64 experimental trials (32 neutral and 32 taboo) and a random number of 3–6 fillers [e.g., *rond clair (light circle)*], unrelated to the experimental priming of the spoonerisms, which sometimes required a verbal response as in Motley and Baars (1976a). These filler pairs were presented in order to avoid predictability of the sound signal. All word pairs were presented for 800 ms in white against a black font with 50 ms interstimulus intervals (fixation crosses). The speech prompt was a sound signal that occurred 270 ms after the target presentation and participants had 2,500 ms to give a verbal response. All trials were randomized for each participant. Participants’ responses were digitally recorded using the computer’s in-built microphone. The audio files were later listened to independently by two experimenters to check for the occurrence of spoonerisms (no differences were recorded between both judges).

In accordance with the original Motley-studies (e.g., Motley and Baars, 1976b; Motley et al., 1981b) as well as with e.g., Möller et al. (2007), Costa et al. (2006), and Hartsuiker et al. (2006), spoonerisms were counted as such when at least one phoneme was exchanged (e.g., *mad bug* → *mad mug* or *mad bug* → *bad bug*). Spoonerisms thus involve both partial spoonerisms (i.e., when only one phoneme was exchanged: e.g., *mad bug* → *mad mug)* and complete spoonerisms (i.e., full exchanges: e.g., *mad bug* → *bad mug).* Verbalization errors were counted when participants gave responses that were unrelated to the priming manipulation (e.g., *mad bug* → *rad bug*). Omissions were counted when participants gave no verbal responses to the target pairs.

#### 2.2.2. The geometrical categorization task

**FIGURE 2:**
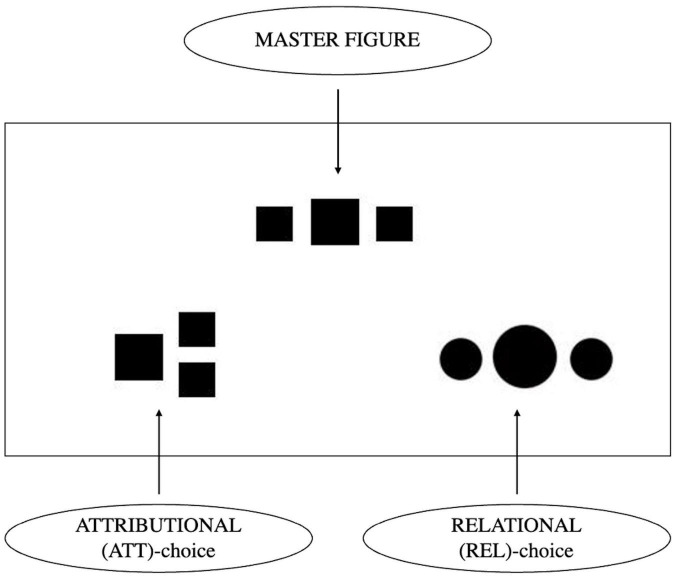
Example of a triad of one item of the Geometrical Categorization Task consisting of a master figure and two target figures. The ATT-target **(left)** consists of the same components as the master figure but in a different configuration and is thought to probe for primary process mentation; the REL-target **(right)** is made up of different components, but these are arranged in the same total configuration and is thought to probe for secondary process mentation.

The GeoCat 1.3 (Brakel et al., 2000; Bazan and Brakel, 2023) is a non-verbal tool which maps preferential mobilization of primary versus secondary processes in terms of similarity between geometrical figures (see Figure 2). Each GeoCat contains 6 items composed of a master figure and two target figures. The participant has to choose the target figure that he considers the most similar to the master figure. There are 4 versions (1A, 1B, 2A, 2B); these versions were randomly attributed to the participants. Series 1A and 1B are identical except that the two lower target figures in the triads are reversed (left-right). The same is true for the series 2A and 2B. This controls the possible effects of lateralization in the target choice. There were no significant differences between the 4 versions in participants’ responses (Kruskal–Wallis *H* = 3.58; *p* = 0.312). The internal consistency of the GeoCat was investigated with Cronbach’s Alpha (α = 0.71).

#### 2.2.3. The PN WordList

The PN WordList (see e.g., Bazan et al., 2019a) consists of 20 French word triads presenting a prime word [e.g., *Note* (*Grade*)] together with a phonologically related target P [e.g., *Tonne* (*Ton*)], which was actually a phonological inverse, and a neutral target N that has no intended relationship, phonological nor semantical, to the prime word [e.g., *Barbe* (*Beard*)]. The participants are asked to choose which of the two target words they think is most similar to the master word. All word triads were randomized for each participant. The internal consistency, investigated with Cronbach’s α, was 0.86.

#### 2.2.4. The Marlowe-Crowne Social Desirability scale

The MCSD (Crowne and Marlowe, 1960; French translation by Vézina, 1989) is a 33-item true/false self-report questionnaire. Given the fact that the MCSD has suffered validity criticisms (see e.g., Leite and Beretvas, 2005), we have investigated its convergent validity in a separate, independent study (*N* = 958) and found significant correlations with a validated French social desirability measure, the DS-36 (Tournois et al., 2000; *r* = 0.67; *p* < 0.001) as well as with the “repressive defensiveness” subscale of the validated *Weinberger Adjustment Inventory* or WAI [French translation by Paget et al. (2010); *r* = 0.60; *p* < 0.001]. We also investigated its divergent validity with the trait-subscale of the State Trait Anxiety Inventory [Spielberger et al., 1970; French translation by Bruchon-Schweitzer and Paulhan (1990)] yielding again predicted results (*r* = −0.32; *p* = < 0.001). The internal consistency Cronbach’s Alpha of the SLIP-MCSD and of the evaluation study-MCSD were 0.49 and 0.75, respectively.

#### 2.2.5. Procedure

The experiments were conducted at the *Université libre de Bruxelles* (ULB). After a short introduction to the laboratory, participants signed an informed consent statement. Participants then were seated in front of a computer screen and received oral instructions. The experimenter was sitting behind so that he was outside of the participants’ field vision. First, they had to complete a brief demographic questionnaire (age, gender, level of French, spoken languages) as well as the GeoCat 1.3. Participants were then invited to do the SLIP-task that actually was presented as a memory experiment for which they were asked to memorize each word pair for a later recall test. This strategy increases the probability of producing spoonerisms (Motley and Baars, 1976a). Our instruction was « *The aim of the present task is to study the memorization of word sequences. For this purpose, word pairs will appear quickly and successively on the screen. Try to remember as many as possible, a memory task will be presented at the end of this task. Please pay attention: some word pairs will be followed by a sound signal; this indicates that you must say aloud the last pair you saw on the screen. Pairs that are not followed by a sound signal should be read silently, internally* ». Immediately after the SLIP-task, participants had 10 min to complete a fake memory test where they were asked to write down all the pairs they had remembered.^3^ Next, they were invited to complete a series of questionnaires including the PN WordList and the MCSD.

## 3. Results

### 3.1. Behavioral results

Participants produced 37 spoonerisms (27 partial and 10 complete), 162 verbalization errors, and 37 omissions: on a total of 3520 trials this corresponds to, respectively, 1% spoonerisms, 4.6% verbalization errors, and 1% omissions. In regard to the number of participants, 22 made spoonerisms, 42 made verbalization errors, and 12 made omissions; on a total of 55 participants this corresponds to, respectively, 40, 76.3, and 21.8%. Taboo slips were produced by 17 participants while neutral slips were produced by 12 participants.

Note that these 37 parapraxes were associated with 11 sequences out of the 32 in the neutral condition and 7 sequences out of 32 in the taboo condition. As concerns the neutral parapraxes, 2 sequences produced 7 out of 16 parapraxes: *pomme roche* (*apple rock, N* = 4) and *pige fil* (*understands wire, N* = 3), the other 9 parapraxes were produced by 9 sequences and 21 sequences produced no parapraxes. As concerns the taboo trials, 7 sequences out of 32 led to spoonerisms, with 3 “star” sequences: *belle pipe* (*nice blowjob, N* = 9), *bite molle* (*limp dick, N* = 4), and *bite chaude* (*warm dick, N* = 4). The 25 other sequences produced no parapraxes.

### 3.2. People make as many “taboo” as “neutral” slips

The descriptive statistics (see Table 1) show that there is no significant difference between the occurrence of taboo and neutral slips (Wilcoxon Signed Ranks test; *p* = 0.348), and even not between taboo and neutral speech errors (Wilcoxon signed Ranks test; *p* = 0.104), even though in absolute numbers there are more taboo slip and speech errors. As concerns the partial slips, there was also no difference between the production of partial neutral or taboo spoonerisms (resp. 13 on 16 and 14 taboo on 21; Wilcoxon Signed Ranks test; *p* = 0.861). Among the partial taboo spoonerisms, Motley et al. (1981a,1982) further distinguished between “safe partials” (e.g., *tool kits* → *cool kits*) and “taboo partials” (e.g., *tool kits* → *tool tits*); there was no difference between the production of safe and taboo partials (resp. 10 and 4, Wilcoxon Signed Ranks test; *p* = 0.153). Interestingly, there were significantly more taboo than neutral omissions (Wilcoxon Signed Ranks test; *p* = 0.005).

**TABLE 1:** Mean ± SEM by participants (*N* = 55) for the total number of slips, speech errors and omissions in both neutral and taboo conditions.

	Total	Neutral	Taboo	*p*
Slips	0.67 ± 0.13 (37 - 1%)	0.29 ± 0.09 (16 - 0.4%)	0.38 ± 0.08 (21 - 0.6%)	0.348
Errors	2.90 ± 0.33 (160 - 4.6%)	1.25 ± 0.17 (69 - 2%)	1.65 ± 0.22 (91 - 2.6%)	0.104
Omissions	0.68 ± 0.28 (37 - 1%)	0.13 ± 0.07 (7 - 0.2%)	0.55 ± 0.22 (30 - 0.8%)	0.005

Total absolute frequencies are given into parentheses; *p*-values for bilateral testing.

Similarly, the category of slips (neutral or taboo) did not explain the occurrence of the slips (Mann–Whitney *U*-test; *p* = 0.488). We also used the empirical tabooness data instead of the *a priori* taboo/neutral classification. The production of slips was also not explained by the tabooness of the intended slips (e.g., *cool tits*; simple linear regression *F*_(1,62)_ = 1.596; *p* = 0.211). As people react upon the eliciting pairs, not necessarily upon the intended taboo puns, we also verified the production of slips in function of the tabooness of the eliciting pairs (e.g., *tool kits*), but this was also not significant (simple linear regression *F*_(1,62)_ = 0.679; *p* = 0.413). These analyses confirm the descriptives: there is no difference in the occurrence frequency of neutral versus taboo slips; we will thus treat all parapraxes as one group.

### 3.3. The production of parapraxes is a defensive process

According to our hypothesis, the production of parapraxes is a defensive process, and therefore we expect to predict their occurrence in function of defensiveness parameters, both primary process (number of ATT on the GeoCat) and secondary process (number of N-choices in the PN WordList). The descriptives of these parameters (as well as of the MCSD) are given in Table 2.

**TABLE 2:** Mean ± SEM for the number of ATT-choices in the GeoCat (on 6), the number of N-choices in the PN WordList (hence N/PN; on 20) and the Marlowe-Crowne Social Desirability scale (on 33); *N* = 55.

	Mean ± SEM	Min	Max
ATT	1.5 ± 0.3	0	6
N/PN	2.0 ± 0.4	0	14
MCSD	17.5 ± 0.5	9	25

When we test this model for all parapraxes, the overall regression was significant: *F*_(2,51)_ = 8.841; *p* < 0.001 with an *R*^2^ = 25.7%. The occurrence of parapraxes is significantly explained by the number of ATT (B = 0.186; *p* = 0.003) and the number of N/PN (B = 0.099; *p* = 0.007).

We also tested the whole model for the omissions and for the verbalization errors, to investigate whether a defensive process is also implied in the omissions and verbalization errors. This was not significant, for omissions (*F* = 0.009; *p* = 0.991) nor for verbalization errors (*F* = 0.522; *p* = 0.596).

### 3.4. Highly defensive people produce more parapraxes than lowly defensive people

**FIGURE 3:**
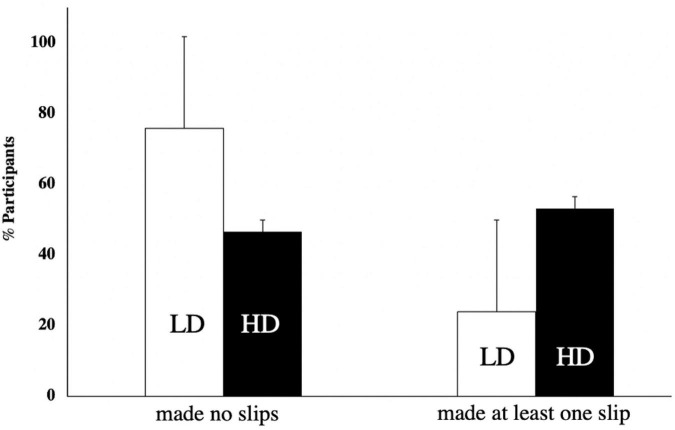
Main behavioral effects: percentage of number of lowly (*N*_total_ = 25) and highly (*N*_total_ = 30) defensive participants who made no slips versus at least one slip (χ^2^_(1)_ = 4.889; *p* = 0.027; the χ^2^ test indicates that the total correlation between “making no or at least one slip” and “being lowly or highly defensive” is significant).

Following Weinberger et al.’s (1979) basis for his “repressor’s” taxonomy as well as Davis and Schwartz (1987) and Furnham et al. (2003) we isolated lowly and highly defensive participants. Following authors such as Kraft (1998), Ringel (1999), Erskine et al. (2007), or Lévesque et al. (2010), participants’ scores were dichotomized at the median (18) to define participants into lowly defensives (LD; *N* = 25; mean_MCSD_ = 14.0 ± 0.5) and highly defensives (HD; *N* = 30; mean_MCSD_ = 20.4 ± 0.4; *p* = 0.001 with LD). If we now predict the occurrence of parapraxes in function of the defensiveness category of the participants, the simple linear regression model is significant: *F_(1,53)_* = 2.884 (*p* = 0.048, unilateral testing; B_MCSD_ = 0.427). Indeed, highly defensive participants produced double as many slips as lowly defensive participants (Mann–Whitney *U*-test = 273; *p* = 0.025, unilateral testing; see Table 3 and Figure 3).

**TABLE 3:** Mean ± SEM for the number of slips, of verbalization errors and of omissions as well as for the number of ATT-choices in the GeoCat (on 6) and of N-choices in the PN WordList (on 20) by participant, for the lowly and highly defensive participants (LD and HD resp.); *p*-values for bilateral testing.

	Total population (*N* = 55)	LD (*N* = 30)	HD (*N* = 25)	*p*
Slips	0.67 ± 0.13 (37)	0.44 ± 0.17 (11)	0.87 ± 0.18 (26)	0.050
Errors	2.90 ± 0.33 (160)	2.32 ± 0.41 (58)	3.40 ± 0.49 (102)	0.133
Omissions	0.68 ± 0.28 (37)	1.08 ± 0.58 (27)	0.33 ± 0.18 (10)	0.292
ATT	1.5 ± 0.3	1.4 ± 0.4	1.6 ± 0.3	0.327
N/PN	2.1 ± 0.4	1.3 ± 0.4	2.8 ± 0.7	0.133

### 3.5. Only highly defensive people mobilize inhibitory-type defenses for the production of parapraxes: Running away from ambiguity, they stumble upon their words

Since the defensive dynamics might differ qualitatively in highly and lowly defensive people, in agreement with our hypotheses, we now test our model in lowly and highly defensives separately.

In Table 3, the descriptives of the defense parameters (ATT and N/PN) are also given for the lowly and highly defensive participants of our study. At this level, there are no significant differences. However, the regression results explaining the factors for the production of parapraxes in function of the defense parameters are different in both populations.

For the lowly defensive participants (*N* = 25), the multiple linear regression model for all parapraxes with the number of ATT and N/PN is non-significant: *F*_(2,21)_ = 1.754; *p* = 0.198. However, when removing the N/PN parameter, the regression with only the ATT-parameter (B = 0.164) is significant: *F*_(1,22)_ = 3.613; *p* = 0.036; *R*^2^ = 14.1% (unilateral testing). For lowly defensive participants, only the primary process predicts the occurrence of parapraxes.

For the highly defensive participants (*N* = 30), the multiple linear regression model for all parapraxes with the number of ATT and N/PN as predictors, shows a significant effect: *F*_(2,27)_ = 6.601; *p* = 0.005; *R*^2^ = 32.8%. Both ATT (B = 0.210, *p* = 0.019) and N/PN (B = 0.112; *p* = 0.011) predictors are significant. Interestingly, in highly defensive participants both primary and secondary process defenses predict the occurrence of parapraxes.

Note that we also compared lowly (LD; *N* = 25) and highly defensive (LD; *N* = 30) participants for the omissions and the verbalization errors. We found that the ATT and N/PN predictors were not significant for the omissions (LD: *F* = 0.307; *p* = 0.739; HD: *F* = 0.083; *p* = 0.920) nor for the verbalization errors (LD: *F* = 0.974; *p* = 0.394; HD: *F* = 0.168; *p* = 0.847)

### 3.6. What about differences between neutral and taboo parapraxes?

Even if our data show that the *a priori* difference between neutral and taboo parapraxes is not reflected in empirical differences in treating these parapraxes, for reasons of comparability with previous studies, we have explored this distinction nevertheless. Highly defensive participants produced significantly more taboo spoonerisms (μ = 0.53 ± 0.68; Mean rank_HD_ = 31.40) than lowly defensive participants (μ = 0.20 ± 0.50; Mean rank_LD_ = 23.92; Mann–Whitney *U*-test; *p* = 0.033). However, for the number of neutral spoonerisms no significant differences between highly (μ = 0.33 ± 0.60; Mean rank_HD_ = 29.30) and lowly defensives (μ = 0.24 ± 0.66; Mean rank_LD_ = 26.44) were found (Mann–Whitney *U*-test; *p* = 0.360).

As concerns the regression results explaining the factors for the production of parapraxes in function of the defense parameters in both populations, for neutral parapraxes, we find the same differences as already found for all the parapraxes; interestingly, nothing comes out of the regression analyses for the taboo parapraxes:

• For the lowly defensive participants (*N* = 25), a simple linear regression *for neutral slips* with ATT as a predictor is significant: *F*_(1,22)_ = 5.968; *p* = 0.023; *R*^2^ = 21.3% with for ATT: B = 0.155. The linear regression in this group *for taboo slips* with ATT is not significant: *F*_(1,22)_ = 0.033; *p* = 0.857 (with for ATT B = 0.010).
• For the highly defensive participants (*N* = 30), the multiple linear regression *for neutral slips* with ATT and N/PN as predictors, shows a significant effect: *F*_(2,27)_ = 6.606; *p* = 0.005; *R*^2^ = 32.9%. Both ATT (B = 0.146; *p* = 0.010) and N/PN (B = 0.062; *p* = 0.022) predictors are significant. The multiple linear regression in this group *for taboo slips* with ATT and N/PN is not significant: *F*_(2,27)_ = 1.507; *p* = 0.240 (with for ATT, B = 0.064; *p* = 0.343; and for N/PN, B = 0.049; *p* = 0.148).

## 4. Discussion

Altogether, 37 parapraxes were produced in this study, by 22 participants out of the 55. Although these 37 parapraxes represent about 1% of the experimental trials,^4^ this number is comparable to other studies having replicated the SLIP technique (e.g., Hartsuiker et al., 2005: 6.5%; Costa et al., 2006: 1.9%; Severens et al., 2011: 2.4%; Wagner-Altendorf et al., 2020:0.7%). The proportion of partial spoonerisms in the total number of spoonerisms is high (more than 70%), but it is comparable to what has been reported in other studies (ca. 47% in Costa et al., 2006 and 68% in Hartsuiker et al., 2006). Interestingly, these frequent partial spoonerisms echo with Freud’s claims on verbal parapraxes, namely that they are « an outcome of a compromise: they constitute a half-success and a half-failure for each of the two intentions; the intention which is being challenged is neither completely suppressed nor, apart from special cases, carried through quite unscathed » (Freud, 1916–1917/1966, p. 66).

As concerns the nature of the parapraxes, the 2 “star” parapraxes responsible for more than 40% of the neutral parapraxes–*pomme roche* (*apple rock*) and *pige fil* (*understands wire*) – show a repetition of the middle vowel that might have facilitated these parapraxes (Motley and Baars, 1976a). Similarly, the fact that/b/and/p/are consonants are close in acoustic and articulatory characteristics has probably facilitated the taboo parapraxis *belle pipe* (*nice blowjob*) responsible in and by itself for more than 40% of the taboo parapraxes. Even if the taboo top 3 sequences are three “penis”-stimuli (next to *belle pipe* there was also *bite molle*–*limp dick* - and *bite chaude*–*warm dick*), which might suggest that the “penis”-meaning acts as an amplifying factor, it should be said, though, that the parapraxes *bite frotte (dick rubs), bande fort (big boner), belle couille (nice nuts), and couille molle (half-sack)* were not produced, weakening this proposition. Therefore, we have no strong indication for the nature of the meaning universally determining the probability of making slips. In conclusion, even if this was not the focus of the present research, it seems that elements concerning the phonological nature of the stimulus material influence the probability of making slips, which is in agreement with linguistic research (Motley and Baars, 1976a). Even if our study investigates what in the personality of the participants makes the tongue slip, we do not expect that personality structure covers the whole (or even the major) part of the variability, leaving room for other factors, including the phonological nature of the stimulus material.

### 4.1. People make as many “taboo” as “neutral” slips

One main finding is that, contrary to Motley et al.’s, recurrent finding that people make less taboo than neutral slips, we did not find such a significant difference between taboo and neutral slips. To take into account that there might be a different understanding (depending on time and place) of what is “taboo,” we measured tabooness in an independent sample of *N* = 120 participants, parallel to the SLIP study. This study confirmed that, on average, the taboo spoonerisms (e.g., *cool tits*) were indeed rated as significantly more taboo than the neutral spoonerisms (e.g., *barn door*). However, tabooness varied considerably, and overlapped partially between *a priori* taboo and *a priori* neutral pairs (the overlap was in the tabooness-range 1.8–2.1). In fact, one taboo spoonerism, *seins mère* (*mother’s boobs*) fell into the range of the “neutral” word pairs (tabooness = 2.11). For this reason, next to using the *a priori* taboo/neutral categories, we also regressed the probability of making a parapraxis upon the tabooness index, but this regression was also not significant. Finally, we did the same exercise with the tabooness of the eliciting pairs, with no significant results. By all means, then, we may say that there were no significant differences between the numbers of taboo and neutral parapraxes.

Severens et al. (2011) also did not find significant results. Both in our study and in Severens et al. (2011) absolute numbers were even *higher* for taboo slips than for neutral slips, but in both studies the difference was not significant. To our knowledge, only Wagner-Altendorf et al. (2020) have replicated Motley et al. (1981a,1982) original results, finding significantly more neutral than taboo slips. Motley et al. (1982, p. 580) had interpreted their findings of fewer taboo slips as an indication for the existence of a prearticulatory editing component that prevents the overt formulation of taboo words on the basis of « social appropriateness ». Having not replicated their results, we do not, however, interpret these findings as a disproof of Motley’s proposed “editor” or “censor”-principles. Indeed, we had initially predicted, following herein Freud’s repeated injunctions (see Introduction), that all slips, even the « apparently simple » (Freud, 1901/1978, p. 83) or « the mildest cases » (Freud, 1901/1978, p. 279), were to be understood as the result of a defensive process, and that the *a priori* difference between “neutral” and taboo is not valid at a singular subjective level.

### 4.2. The production of parapraxes is a defensive process

Moreover, our results are in line with Freud’s hypothesis that in general parapraxes are to be understood as a result of defensive mechanisms (see Introduction: Freud, 1901/1978, p. 279). With our two parameters, the ATT in the GeoCat, which is thought to “catch” primary process mentation, and the N/PN parameter, which is supposed to “catch” a secondary process defensive move against linguistic ambiguity, our model captures a big fourth of the variability in the production of parapraxes in a significant way (with both parameters being significant). In other words, both an increase in primary process mentation and an increase in defensive avoidance of language ambiguity significantly predict the occurrence of parapraxes, confirming our first hypothesis. Furthermore, these ATT and N/PN parameters did not significantly predict the number of omissions and verbalization errors. Therefore, only parapraxes lend themselves to analysis and interpretation in terms of mental categories confirming the Freudian position that slips of the tongue are not simply “system glitches” but subjectively intentional mental phenomena.

We are, of course, not overly amazed to catch only a quarter of the variance of the parapraxes production. Our expectation is that the by chance-correspondence of the presented meanings with the singularly important meanings of the specific subject will actually catch the big chunk of the variance. Our bet was that, beyond the singularity of the meanings, universal formal logics do play a role in explaining or predicting the probability of producing parapraxes. Indeed, it appears that not everything concerning our mental productions is a question of the meanings which inhabit our subjective life. There are formal organization logics which structure this world of meanings and, moreover, which may structure them differently according to personality. For example, Bergeret, (1974, p. 46) has described a personality in terms of a « rather invariant reciprocal play of the primary and secondary processes ». For all these reasons, following Freud, clinical experience, and in accordance with others, defensiveness and its articulation as a (differential) combination of primary and secondary process mentation are used here as the key principles to map these different organizations.

### 4.3. Highly defensive people produce more parapraxes than lowly defensive people

We distinguished two populations, lowly and highly defensive participants, in the general population based upon their results on the Marlowe Crowne Social Desirability scale. We have argued that although this scale is originally designed to measure social desirability, it has almost directly and consistently since been used by the original authors (Crowne and Marlowe, 1964) and by others (e.g., Weinberger et al., 1979; Weinberger, 1990; Eysenck and Van Berkum, 1992; Mann and James, 1998), as a measure of defensiveness. Still, as such it has remained a controversial measurement tool because of its ambiguous factorial structure (e.g., Leite and Beretvas, 2005). For this reason, we have measured, in a separate population, its convergent and divergent validity with another validated tool for social desirability, as well as with a validated “repressive defensiveness” scale and with a validated anxiety inventory, all yielding coherent results (Bruchon-Schweitzer and Paulhan, 1990; Tournois et al., 2000; Paget et al., 2010). Nevertheless, our Cronbach’s Alpha in the present study was not good, and this is one of the limitations of this study. The low *N* in the present study (55) probably explains this low Cronbach’s Alpha, as our parallel variable evaluation study for the MCSD with a much larger population yielded a good Cronbach’s Alpha. In this respect, the eigenvalue of the first factor in the Principal Component Analysis (PCA) of the SLIP-MCSD was 3.6 and Yurdugül (2008) underscore that with only one factor with an eigenvalue between 3 and 6, a sample of *at least 100* is needed to reliably calculate the Cronbach’s Alpha. Furthermore, in the present study, we only used the SLIP-MCSD results to divide our population in lowly and highly defensive participants and our criterion (the median of 18) is comparable in absolute numbers to the criterion used in other studies: e.g., Weinberger et al. (1979), Fuller and Conner (1990), and Weinberger and Davidson (1994) divided their population at resp. 17, 18, and 19, using the upper quartile as a criterion. Finally, even with this weak Cronbach’s Alpha, the dichotomic categorization on the MCSD significantly predicted the occurrence of parapraxes in function of the defensiveness category, empirically confirming the relevance of this distinction in the population. For these reasons, we think that our categories of lowly and highly defensive participants are valid categories. Our results now show that highly defensive parameters make almost double as many parapraxes compared to lowly defensive parameters (see Figure 1), but do *not* make more speech errors or more omissions. This confirms the link between defenses and parapraxes. To further explain this result, we have investigated the primary and secondary process logics linked to the occurrence of parapraxes in each population.

### 4.4. Only highly defensive people mobilize inhibitory-type defenses for the production of parapraxes: Running away from ambiguity, they stumble upon their words

Our results show that the occurrence of parapraxes in lowly defensive parameters is only significantly explained by the ATT primary process parameter and with a low *R*^2^ (14%). The N/PN parameter does not contribute to explaining the variance in the occurrence of parapraxes. In highly defensives, however, the model with both parameters explains almost a third of the variance, with both the primary and secondary process parameter being significant.

The low contribution in explained variance in the lowly defensive parameters does not mean, in our opinion, that their defenses are necessarily low. As said in the Introduction, we consider, with Freud, that the ontological nature of mental processes is defensive in essence. In the primary process logic, the defense consists in directly associatively discharging the word (pairs) that have gathered a high tension - this is, without this tension being first built up to stocked excitation by means of inhibition. To defend against the excitement caused by the possibility of saying *cool tits* when reading *tool kits*, one simply directly says *cool tits*. This direct and transparent way of dealing with uptight topics is in colloquial language, paradoxically, sometimes qualified as “non-defensive,” especially in its opposition with inhibitory, restrictive defenses. This should not obscure the basic fact, however, that it also is defensive. The obsessional preoccupation with or projection of the same topic over and again, it being thereby positively present as Erdelyi (2006) would say, shows in its exaggeration that primary process directness is, in essence, also defensive. However, we think we could not catch this primary process logic in an effective way due to the fact that we might not have an adapted measurement tool for primary process linguistic mechanisms.

In the highly defensive participants, in contrast, we succeeded in capturing a good part of the explained variance with our two parameters. However, it is important to point out that the distinction between lowly and highly defensives was made on the basis of the extent to which participants were inclined *not to acknowledge* undesirable social behavior. In other words, highly defensive participants are thought to specifically have high defenses of the second category, the inhibitory category - this is, the category linked by us to the secondary process. This means that our findings might at first sight look somewhat circular: we selected participants with high inhibitory defenses and then find that indeed their parapraxes are explained by these inhibitory defenses. However, we must remember that our measure for “these inhibitory defenses” is quite radically different from the MCSD-personality questionnaire, as it concerns a very basic linguistic similarity preference (between a phonologically similar and a non-similar choice). It is therefore actually rather revealing that participants deemed defensive on the basis of the MCSD are also making more parapraxes in correlation with the avoidance of language ambiguity. In other words still, it is remarkable to find here empirical evidence for the idea that psychodynamic defenses show up as a specific manner of processing language (see also Lacan, 1966), a manner which we may catch by the phrase: with increasing defensiveness, we run away from ambiguity and stumble upon our words. Indeed, the avoidance of subliminal ambiguity was found before (see Bazan et al., 2019a); moreover, here this avoidance is linked to a higher probability to make parapraxes, i.e., to stumble upon words.

In summary, defensive dynamics are both similar and different in lowly and highly defensive parameters. They are similar in that primary process defenses might possibly be seen as baseline default defenses, but different in that highly defensive participants mobilize an additional mechanism, which, as we propose, is inhibitory in nature and is secondary process in type.

### 4.5. What about differences between neutral and taboo parapraxes?

Even if the difference between neutral and taboo parapraxes is an artificial, non-empirically confirmed distinction, when we apply these *a priori* categories, we find that highly defensive participants produce more than double the number of taboo parapraxes produced by lowly defensive participants, while they did not produce significantly more neutral parapraxes, even if the number were higher in absolute terms. Interestingly, understanding the production of taboo parapraxes selectively in terms of primary and secondary processes does not work: in lowly defensive parameters, where the model only takes the primary process, results are nowhere and in highly defensive participants, where the model takes both primary and secondary process parameters, the results are not significant. In contrast, the production of neutral parapraxes in lowly and highly defensive participants gives results in line with those for the total group of parapraxes: in lowly defensives the model is significant with the ATT accounting for a good 20% of the explained variance; in highly defensives the significant model accounts for a third of the variance, with both ATT and N/PN being significant.

This, then, brings us to the following speculation: indeed, the universally taboo parapraxes form a distinct group, and indeed, what distinguishes them from the universal category of “neutral” parapraxes, is that they are as a group more subject to an editing process. However this editing process pointed out by Motley et al. (1981a,1982) is, in our view, primary nor secondary process-type. This is, it is not an *unconscious* defense type; it is not repression, but a (pre-)conscious *suppression* mechanism. Some other results point in that direction. Indeed, impressively, in our parallel study on the tabooness of the word pairs implied in the SLIP-task, - namely, the spoonerism eliciting pairs and the spoonerisms themselves - subjects rated taboo eliciting pairs (e.g., *tool kits*) as being more taboo than neutral eliciting pairs (e.g., *darn bore*). We propose that these results show that people are capable of anticipating taboo slips. This might suggest that there is a preconscious intuition of the taboo outcome of words that are nevertheless neutral at first sight. The difference between suppression and repression is made explicit in a superb way by Freud (1901/1978) in his explanation of the forgetting of the name “Signorelli.” The first substitute, which comes to his mind, instead of Signorelli, is *Botticelli*. He explains this substitution by suggesting that the mental cathexis of “signor,” under inhibition, has by the way of its translation to “Herr,” followed by the word-bridge “Herzegowina und Bosnien,” migrated to the syllable “Bo,” which, together with the uninhibited syllable “elli,” leads to Botticelli. Crucially, he indicates that he had consciously swallowed a phrase he was about to say to his travel companion and which started as “*Herr*, was ist da zu zagen” (« *Sir*, what is there to be said? »). He remembered having done so since it referred to inappropriate sexual content. He did so well in withholding this, that the inhibition spilled over to the associated fragments and he also inhibited the associated semantic and phonological variants, including the Italian translation of *Herr*, *signor*, and the phonologically associated *Signorelli*. But since the move is consciously remembered, this is suppression, not repression. However, another substitute comes to his mind, *Boltraffio*. Associating upon this substitute, Freud, (1901/1978, p. 3) now remembers that one of his patients over whom he « had taken a great deal of trouble », had committed suicide and that he came to know this information when he was in Trafoi. It makes sense that the chain of associations, drifting upon the general theme “death and sex,” had also activated *Trafoi*, which, inheriting the highly cathected “Bo,” could find discharge, disguised as “Boltraffio.” However, Freud had no conscious recollection whatsoever that this patient had come to his mind and only reconstructed this probability in the aftermath on the basis of the substitute word. This, then, indicates that the “Trafoi”-associations were properly repressed and Boltraffio is the return-of-the-repressed.

What we propose, then, is that the Motley prearticulatory editor indeed intercepts taboo parapraxes in such a way that there are significantly more taboo-parapraxes that were swallowed in a final editing process than neutral parapraxes. We propose that this “swallowing” is a late censorship, proximal to utterance and independent of the intimate personality organization, and therefore not explainable in terms of primary process and secondary process inhibition, as seen in our results. Two independent results give more weight to this hypothesis. First, the number of omissions in taboo target pairs were more than five times the number of omissions in neutral target pairs (see Table 1), suggesting people might more often “swallow” a taboo parapraxis they are about to make. Also, the number of omissions was not explainable in terms of primary and secondary processes mentation as the model was not significant.^5^ Second, there was no significant difference in the number of omissions between lowly and highly defensive participants (in absolute numbers, the highly defensives had even less), indicating that indeed the “swallowing” was not an unconscious defense move, explainable in terms of primary process and secondary process inhibition, but indeed an editing process of another nature, comparable in psychoanalytic terms to conscious or preconscious suppression. It might also be called a “cognitive correction” which happens quite independently of personality (Shevrin, 1992).

This interpretation would also fit nicely with the other SLIP-studies, including a number of more recent neuro-imaging results. First, let’s recall Motley et al. (1981a,1982); p. 9, p. 580 editor is described as « prearticulatory editing on the basis of social appropriateness », much along what Freud, (1901/1978, p. 3) himself recount as concerns his swallowing of the « *Herr*, was ist da zu zagen »-anecdote: « I suppressed my account of this characteristic trait, since I did not want to allude to the topic in a conversation with a stranger » (Freud, 1901/1978, p. 3). As said, we propose that the censorship which is at play here is not the censorship between the system Ucs and conscious processing (namely, repression) but rather, the second censorship, the one between the Pcs and the Cs: « a mental act commonly goes through two phases, between which is interposed a kind of testing process (censorship). In the first phase the mental act is unconscious and belongs to the system Ucs; if upon the scrutiny of the censorship it is rejected, it is not allowed to pass into the second phase; it is then said to be “repressed” and must remain unconscious. If, however, it passes this scrutiny, it enters upon the second phase and thenceforth belongs to the second system, which we will call the Cs. But the fact that is so belongs does not unequivocally determine its relations to consciousness. It is not yet conscious, but it is certainly *capable of entering consciousnes*s, (…) that is, it can now, *without any special resistance* and given certain conditions, become the object of consciousness. In consideration of this capacity to become conscious we also call the system Cs the “*preconscious*” » (Freud, 1915/1963, pp. 122–123; Italics added). We propose that a number of slips, having passed the censorship of repression, and being readied to be discharged, i.e., executed or articulated, are halted at that stage: to cite Freud, (1915/1963, p. 123), even if they were « capable of entering consciousness », they encountered « special resistance », and were censored from the system Cs; this particular resistance would then precisely be Motley et al.’s socially motivated prearticulatory editor.

Severens et al. (2011) found that, slightly after being confronted with the speech prompt, participants showed a larger negative brain wave in the taboo condition compared to the neutral condition, even if they produced no spoonerisms (see also Wagner-Altendorf et al., 2020). The authors interpreted this as evidence that taboo errors « are formed, detected, and corrected internally » and as « the first direct evidence that covert editing of speech exists » (Severens et al., 2011, pp. 1256–1257) which might also constitute evidence that *preconscious* editing in speech exists. In 2012, the same authors show that the inhibition of taboo words activates the right inferior frontal gyrus (rIFG) - a region which might implement neural inhibition of manual (Aron et al., 2003, 2007; Chambers et al., 2006) and verbal responses (Xue et al., 2008). Furthermore, the rIFG has been previously associated with *externally* triggered inhibition (see Severens et al., 2012; e.g., control of risky behavior and delayed gratification as well as emotion regulation). Severens et al., (2012, p. 431) comment:« This finding strongly suggests that external social rules become internalized and act as a stop-signal » and refer to the fact that it is through education and socialization, i.e., through external signals, that we have learned to inhibit inappropriate behavior, as well as not to utter taboo words.

On the other hand, inhibition that is not guided by an external cue but rather internally guided (endogenous self-control), has been demonstrated to involve the dorsal fronto-median cortex (dFMC; Brass and Haggard, 2007, 2008; Kühn et al., 2009). As pointed out by Severens et al., (2012, p. 431), it might be « very crucial to distinguish endogenous from externally guided inhibition » conceptually, confirming also their neuroanatomical distinction. However, we disagree that « external guided inhibition is not a result of deliberation but is rather triggered by the environment » while « by contrast, endogenous self-control is related to a deliberate decision » (Severens et al., 2012, p. 431). We think that *any instance* of inhibition is a result of (subjective) deliberation. When it comes to « externally inhibited » behavior, this deliberation is relatively easy to access consciously, and might correspond to the censorship between the systems Pcs and Cs in the psychoanalytic model. Endogenous self-control, on the other hand, concerns control over internally motivated action, of which a large part is conscious but of which, we propose, another (considerable) part remains unconscious. Freudian repression is supposed to have this characteristic that it happens without any conscious awareness whatsoever and that is very difficult to become aware of, and therefore also to record in experimental set-ups. As seen, it often involves logical suppositions and *post hoc* reconstructions similar to the “Boltraffio/Trafoi”-reconstruction. The self-control decision not to act upon certain endogenous urges, even if deliberate, might thus happen completely unconsciously (which then would constitute an instance of repression) while still activating (dFMC) brain areas involved in decision making (Brass and Haggard, 2007, 2008; Kühn et al., 2009).

These different considerations suggest that the Motley prearticulatory editor remains an *external* instance of inhibition. This external nature pertains to the actual role of others, of the social realm, of the ones we relate to in the very moment - see also the different ways this “actual other” was operationalized in different SLIP-studies [e.g., a sexually provocative experimentor, in Motley and Baars (1978)]. In our view, endogenous self-control pertains to the intimate realm of singularly specific meanings, out of which the endogenously motivated actions spring. As the prearticulatory editor guarantees the social norms, even when internalized, it remains the internal representative of these social norms; it does not relate to the intimate realm of singularly specific meanings of the subject. Concretely, independently of one’s life story *belle pipe* (*nice blowjob*) is taboo, but if you had a nasty experience in a *lighthouse* then *mauve phare* (*purple lighthouse*) might be to you specifically very taboo. In our results, we saw how the slips as a total group, and specifically the so-called “neutral slips,” were to a certain extent predictable in terms of the personality-specific mix of primary process and secondary process inhibition. To the contrary, the taboo spoonerisms did not form anything like a coherent group in relation to these mental categories - in other words, they did not as a group acquire a mentally intimate taboo significance (independently of the personality structure of the participants), and for this reason were not as a group more subject to repression than other slips.

However, given the higher number of taboo parapraxes in highly defensive participants (ca. 2.65 times the number in lowly defensives), we must assume that even if a number of taboo parapraxes were “luckily” intercepted in time - this is, before utterance - still a number escaped vigilance, and that this number was higher in the highly defensives. It would be logical to think that conflictual themes are under higher pressure in highly defensives, so that when the opportunity to express these themes arises - as in the SLIP task - their tongue tends to slip more, giving them an opportunity to release some mental pressure and to avoid tension accumulation. This, then, is precisely Freud’s (1915/1963) « return-of- the-repressed ». It also means that, next to being more regulated by socially internalized prohibitory rules, taboo stimuli also must have a higher probability to be conflictual, even if only so for the highly defensive participants.

Our study shows experimental evidence to support a psychodynamic explanatory model for the production of parapraxes and more widely that psychodynamic (Freudian) phenomena lend themselves in a refutable way to experimental research. Together with previous experimental studies on subliminal language ambiguity (Bazan et al., 2019a) and on the unwitting resolution of rebuses (Olyff and Bazan, 2023), imbedded in a clinically inspired theoretical model (e.g., Bazan, 2007, Bazan, 2012; Bazan and Snodgrass, 2012; Bazan et al., 2021), this study contributes to the critical development of the psychoanalytic corpus and its fruitful integration into the scientific corpus of adjacent disciplines, such as cognitive neuroscience, psycho- and neurolinguistics.

## 5. Limitations

One of the limitations we already mentioned and discussed (see higher) is the low value for the Cronbach’s Alpha of the MCSD in the present study. Another limitation is that the parallel study on the subjective evaluations for the tabooness of the word pairs presented in the SLIP-task were done by external participants.

## 6. Conclusion

In conclusion, our research in 55 French speaking participants with 32 taboo and 32 neutral parapraxes, administered through the SLIP method (e.g., Motley and Baars, 1976a) shows that, contrary to previous results, people do not make more taboo than neutral parapraxes and that, in line with Freud’s ideas, all parapraxes can be partially explained in terms of defenses, both of the elaborative primary process and of the inhibitory secondary process kind (Erdelyi, 2006). Splitting up the population in lowly and highly defensives proved productive as it shows that only in highly defensive people the production of parapraxes also involved a secondary process type of defense against language ambiguity. In other words, the more we run away from language ambiguity, the more we stumble upon words. This kind of findings contribute to psychoanalytic knowledge by enabling to experimentally back-up a Freudian model of repression and of return of the repressed. At the same time, our results corroborate the existence of the prearticulatory editor for the taboo words, but situate it at an external locus of control, independent of the intimate singular mental life, and comparable to the censorship between the systems Preconscious and Conscious in a metapsychological model.

## Data availability statement

The raw data supporting the conclusions of this article will be made available by the authors, without undue reservation.

## Ethics statement

The studies involving human participants were reviewed and approved by the “Comité d’Avis Éthique de la Faculté des Sciences Psychologiques et de l’Éducation” of the Université Libre de Bruxelles. The patients/participants provided their written informed consent to participate in this study.

## Author contributions

AB had the idea for the research. LT and LP created the French SLIP-task stimuli, operationalized the protocol on PsychoPy, conducted the experiments, and carried out the data analysis. AB, SD, and GO provided supervision at all stages of the research. LT and AB wrote the manuscript. All authors contributed to the article and approved the submitted version.

## Footnotes

1 Bazan, A. (submitted). Primary and secondary process mentation and the pertinence of the Freudian model for modern science.

2 Language is supposed to be full of such innuendo’s (see also Freud, 1901/1978, p. 239) but (because of repression), we mostly don’t hear them. The psychoanalyst Gauthier Lafaye tells a story about one of his patients whose father had left the house when she was a kid. During a session, the woman tells « Ma mère n’était pas parvenue » (« My mother did not succeed in ») but pauses when she says « par-venue », leading the analyst to hear another meaning « papa revenue » (« daddy has come back »). When the analyst simply repeats « papa r’venu » this opens a new line of emotional associations, leading the patient to express, for the first time, her grief about the loss of her father. Independently from psychoanalysis, the psychologist Robert Haskell has proposed that the thickness of enunciation in a social exchange also includes a layer which is not consciously experienced, which is articulated at the level of phonological polysemy and which deals with the relationship between the speakers. He has even developed a methodology to track such dual meanings in speech (Haskell, 2003). His matrix proposes in a particular exchange, for example, the phonemic reading of the word group « The stuffy nose » as « The stuff he knows », and together with other example, he suggests that even in apparently innocuous descriptions, peoples express their (unconscious) preoccupations with their mutual relationships of power and seduction.

3 As concerns the fake memory tests, results were not exploitable. The results were very variable: some participants remembered word pairs while others only remembered single words which could have appeared in different trials, the number of word (pairs) recalled by the participants was very variable (between 1 and 15) and some participants remembered words that were not part of the stimuli that were presented. These results were not surprising given the large amount of words presented during the SLIP-task. When counting only word couples, there were not enough results, with a lot of missing results in participants, to make statistical analyses. We also did not notice any remarkable qualitative results (e.g., parapraxes emerging or recurring in the memory test).

4 We discussed this point with Michael Motley and in a personal communication (April 2020), he explained that « we had trouble ourselves when trying with presentation *via* computer. The memory drum was slightly noisy and had an audible rhythm that created a metronome effect. I don’t know for certain, but I think that the “ka-chunk” sound was an important part of the SLIP technique ». This might be true. However, even if the memory drum might have enabled more slips, the laboratory conditions are close to the slips occurring naturally and do not prevent from doing statistical analyses.

5 A total of 12 out of 55 participants actually made omissions. Anecdotally, 13 of the 37 total omissions were made by only one participant. These omissions appeared shortly after the participant made a taboo parapraxis - *belle pipe* (*nice blowjob*) - and 10 of the 13 omissions indeed concerned taboo eliciting trials. This confirms the idea of conscious swallowing, here probably after being alarmed by the own first (taboo) parapraxis.
